# Mechanical activation with Easy Clean device enhanced organic tissue removal from simulated internal root resorption in a laboratory evaluation

**DOI:** 10.1186/s12903-023-03122-8

**Published:** 2023-06-12

**Authors:** Laise Pena Braga Monteiro, Sérgio Emilio Miranda de Sousa, Roberta Fonseca de Castro, Emmanuel João Nogueira Leal da Silva, Juliana Melo da Silva Brandão

**Affiliations:** 1 Department of Endodontics, Federal University of Pará, Rua Augusto Correa, 1, Guamá, Belém, PA 66075-110 Brazil; 2 Department of Endodontics, Fluminense Federal University, Niterói, Rio de Janeiro, Brazil; 3 Department of Endodontics, State University of Rio de Janeiro, Rio de Janeiro, Rio de Janeiro, Brazil; 4 Department of Endodontics, Grande Rio University, Duque de Caxias, Rio de Janeiro, Brazil

**Keywords:** Endodontics, Root resorption, Root canal preparation, Root canal therapy

## Abstract

**Background:**

Considering the irregular shape of the root canal, removing inflamed pulp and granulation tissue completely from internal resorption cavities during chemomechanical preparation can be challenging. This study aimed to evaluate the effectiveness of passive ultrasonic irrigation (PUI) compared to mechanical activation with Easy Clean in the removal of organic tissue from simulated areas of internal root resorption.

**Methods:**

The root canals of 72 extracted single-rooted teeth with oval canals were instrumented with Reciproc R25 instruments. After root canal preparations, the specimens were split longitudinally, and semicircular cavities were prepared using a round bur on each half of the roots. Samples obtained from bovine muscle tissue were weighed and adapted into semicircular cavities. The roots were reassembled and joined, and the teeth were divided into six groups (*n* = 12) according to the irrigation protocol: Sodium hypochlorite (NaOCl) without activation; NaOCl + PUI; NaOCl + Easy Clean; distilled water without activation; distilled water + PUI; and distilled water + Easy Clean. After irrigation protocols, the teeth were disassembled, and the remaining organic tissue was weighed. Data were analyzed by two-way ANOVA and Tukey’s post hoc test (*p* < 0.05).

**Results:**

None of the experimental protocols totally removed the bovine tissue from simulated cavities. Tissue weight reduction was significantly affected by the activation method (*p* < 0.05) and by irrigation solution (*p* < 0.05). Groups with NaOCl irrigation presented higher tissue weight loss when compared to distilled water, for all irrigation methods (*p* < 0.05). The use of Easy Clean resulted in the greatest tissue weight loss (42.0%—Distilled water/45.5%- NaOCl) compared to those of PUI (33.3%—Distilled water/37.7%- NaOCl) and no activation (33.4%—Distilled water/38.8%- NaOCl) (*p* < 0.05). However, no differences were observed between PUI and no activation groups (*p* > 0.05).

**Conclusions:**

Mechanical activation with Easy Clean enhanced organic tissue removal from simulated internal resorption more effectively than PUI. Easy Clean for agitation of the irrigating solution is effective in removing simulated organic tissues from artificial internal resorption cavities, being an alternative to the use of PUI.

## Background

Osteoclastic cell activity causes loss of dental hard tissues, resulting in root resorption. This pathology is classified as internal or external, according to the resorptive lesion location [1]. Internal resorption starts within the root canal, usually caused by chronic infection or trauma, and tends to be asymptomatic [1, 2]. Premature loss of affected teeth may occur if root canal treatment is not performed.

Sodium hypochlorite (NaOCl) is the most widely used irrigation solution in cases of internal resorption, mainly due to its antimicrobial property and tissue dissolution capacity [3]. However, its application by conventional irrigation techniques may not be effective within such anatomically complex areas [4, 5]. Therefore, new agitation methods have been sought to optimize tissue dissolution and irrigation. Passive ultrasonic irrigation (PUI) has been widely used for the agitation of irrigating solutions, and this system induces the formation of cavitation and acoustic waves [6]. The ultrasonic tip is inserted into the root canal and activates the solution through ultrasonic energy, improving the cleaning ability and irrigant properties [7]. Several studies have demonstrated their effectiveness in cleaning complex anatomical areas, improving the reduction of pulp tissue remnants and hard tissue debris and calcium hydroxide paste removal [8–11].

Easy Clean (Bassi Endo, Belo Horizonte, MG, Brazil) consists of an acrylonitrile butadiene styrene plastic instrument with an aircraft wing-shaped cross section and a tip 25 and 0.04 taper. This device cleans by (i) agitating the irrigating solution and (ii) during the friction of its blades inside the root canal. This system was shown to efficiently penetrate the irrigating solution into simulated lateral canals and showed good results in smear layer removal, cleanness efficacy, [8, 11–18] reduction of *Enterococcus faecalis* [19] and sealer penetration [20]. Considering the irregular shape of the root canal, removing inflamed pulp and granulation tissue completely from internal resorption cavities during chemomechanical preparation can be challenging. An additional resource, such as Easy Clean may be helpful in such cases. In fact, a previous study using semicircular cavities, to simulate internal resorption, and ground bovine muscle tissue adapted to them, showed that irrigant activation with PUI and XP-Endo Finisher improved the organic tissue removal when compared to non-activated irrigation [21]. However, up to now, no study investigated the efficacy of Easy Clean device in the removal of organic tissue in the internal root resorption cavities.

Therefore, the aim of this study was to investigate the effectiveness of Easy Clean and PUI used during final irrigation protocols on the removal of organic tissue from simulated internal resorption cavities in root canals. The null hypothesis tested was that there is no difference in the organic tissue removal when Easy Clean or PUI are used to active irrigant solution.

## Methods

The manuscript of this laboratory study has been written according to Preferred Reporting Items for Laboratory studies in Endodontology (PRILE) 2021 guidelines [22].

### Simple size estimation

An a priori ANOVA (fixed effects, omnibus, one-way) was selected from the F test family in G*Power 3.1.7 software for Windows (Heinrich Heine, Universität Düsseldorf, Germany). The effect size (= 0.71) was determined through a previous study with a similar methodology [22]. An alpha-type error of 0.05 and power beta of 0.95 were also specified. A total of 72 samples (*n* = 12, per group) were indicated as ideal for identifying significant differences.

### Specimen selection and preparation

After approval from the local ethics committee (protocol 3.693.501/ Federal University of Pará), freshly extracted single-rooted teeth were selected. Teeth with caries, root cracks, resorptions and immature apex were excluded. Mesiodistal and buccolingual radiographs were taken with the aid of a digital sensor (New IDA; Dabi Atlante, Ribeirão Preto, Brazil) to select only teeth with a single root canal and to categorize them as oval or circular-shaped canals, based on De-Deus et al*.* methodology [23]. The space corresponding to the root canal lumen was measured 5 mm from the apex; the canals were classified as oval-shaped when the mesiodistal diameter was 2.5 times larger than the buccolingual diameter. In addition, only root canals with an initial apical size equivalent to a size 15 K-file were selected.

The crowns were removed by using sterile diamond disks under water cooling to standardize root lengths as 14 mm. After access cavity preparation, the working length (WL) was determined 1 mm short of the apical foramen using a size 15 K-file (Dentsply Sirona Endodontics, Ballaigues, Switzerland). All the root canals were instrumented using Reciproc R25 (25/0.08v) instrument (VDW, Munich, Germany), according to the manufacturer instruction. Irrigation was performed using 2.5% NaOCl solution delivered with syringe and a 29-gauge needle (NaviTip; Ultradent, South Jordan, UT, USA). Final irrigation was performed using 17% ethylenediaminetetraacetic acid (EDTA) for 1 min, then the canal was washed using distilled water and dried with paper points.

**Fig. 1:**
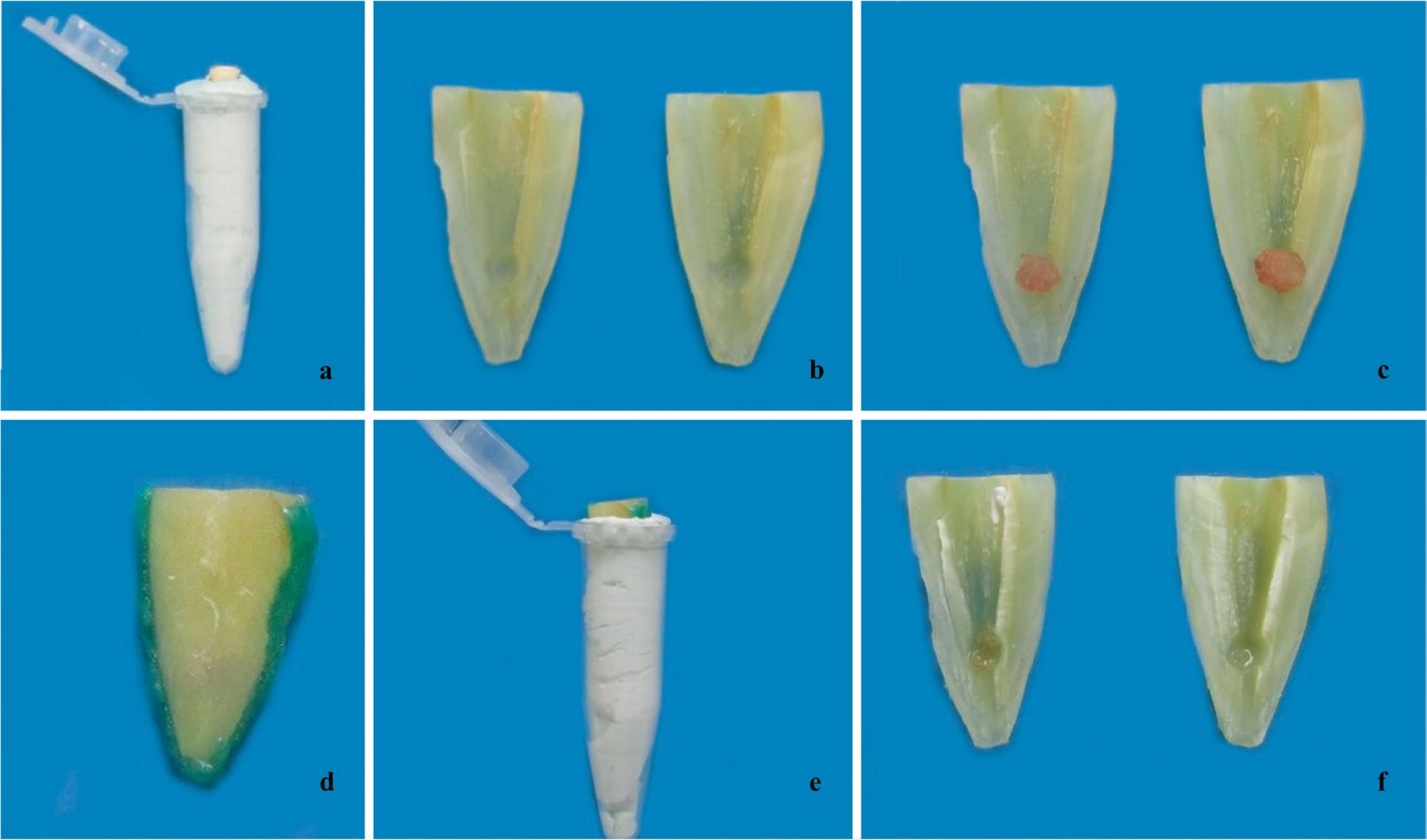
Images of the specimens before and after the irrigation protocols. **a** Specimens embedded in silicone impression material in Eppendorf tubes **b** Teeth split into two halves with simulated cavities **c**) Sample from bovine muscle tissue adapted into the two semicircular cavities of each root **d**) The root halves, including tissue samples, were joined using a gingival barrier **e**) The root halves were reassembled on an Eppendorf tube with silicone **f**) Remaining bovine tissue inside the resorption cavities after the solution activation protocol

The test apparatus was prepared as described by Topçuoğlu et al*.* [24] Samples were embedded in silicone impression material (Coltene/Whaledent, Altstätten, Switzerland) placed in Eppendorf tubes (Fig. 1a). After setting silicone, the specimens were removed, and longitudinal grooves were prepared alongside the roots on buccal and lingual surfaces. The teeth were split into two halves along their long axis with a chisel and hammer. The lengths of the halves were measured by digital calipers. Simulated cavities with 0.8-mm depth and 1.6-mm diameter were prepared with a round bur at the level 5 mm above the anatomic apex of the two halves (Fig. 1b).

Seventy-two samples were obtained from bovine muscle tissue used by the food industry and were adapted into the two semicircular cavities of each root. Then, they were replaced and weighed 3 times using a precision balance with an accuracy of 0.0001 g (Bel Engineering, Monza, Italy). The arithmetic mean of three measurements was calculated and recorded as the baseline value. Then, the preweighed tissue samples were placed again in the semicircular cavities (Fig. 1c). The root halves, including tissue samples, were joined using a gingival barrier (Opal Dam; Ultradent) (Fig. 1d) and reassembled on an Eppendorf tube with silicone (Fig. 1e). Standardized high resolution images of the specimens were taken before and after the protocols irrigation (Canon EOS M50, Tokyo, Japan). The reassembled roots were randomly divided into 6 groups as follows (*n* = 12).

#### NaOCl without activation

The root canals were irrigated with 3 mL 2.5% NaOCl using a syringe and Navitip 29-G irrigation needle (Ultradent) for 1 min without activation. The tip of the needle was placed 2 mm short of the apex. The process was repeated, resulting in 2 min of total irrigation.

#### NaOCl activation with PUI

The root canals were irrigated with 3 mL 2.5% NaOCl, which was activated for 1 min using an ultrasonic tip (E1, Helse, Santa Rosa do Viterbo, São Paulo, Brazil) mounted on an ultrasonic unit (Jet Sonic; Gnatus, São Paulo, Brazil) at a frequency of 30,000 Hz. The ultrasonic tip was placed 2 mm short of the apex. After 1 min, 3 mL freshly prepared irrigation solution was introduced into the root canals, and the process was repeated, resulting in 2 min of total activation.

#### NaOCl activation with Easy Clean

The procedure was similar to those used in the PUI group, but activation was performed using the Easy Clean instrument coupled to the counter angle and operated with a micromotor at approximately 20,000 rotations per minute (KaVo Kerr Group, Charlotte, NC, USA).

#### Distilled water without activation

The root canals were irrigated manually with distilled water in the same manner as NaOCl without activation.

#### Distilled water activation with PUI

The root canals were irrigated with distilled water and activated in the same manner as the NaOCl activation with PUI group.

#### Distilled water activation with Easy Clean

In the Easy Clean group, root canals were irrigated with distilled water and activated in the same manner as the NaOCl activation with Easy Clean group.

**Fig. 2:**
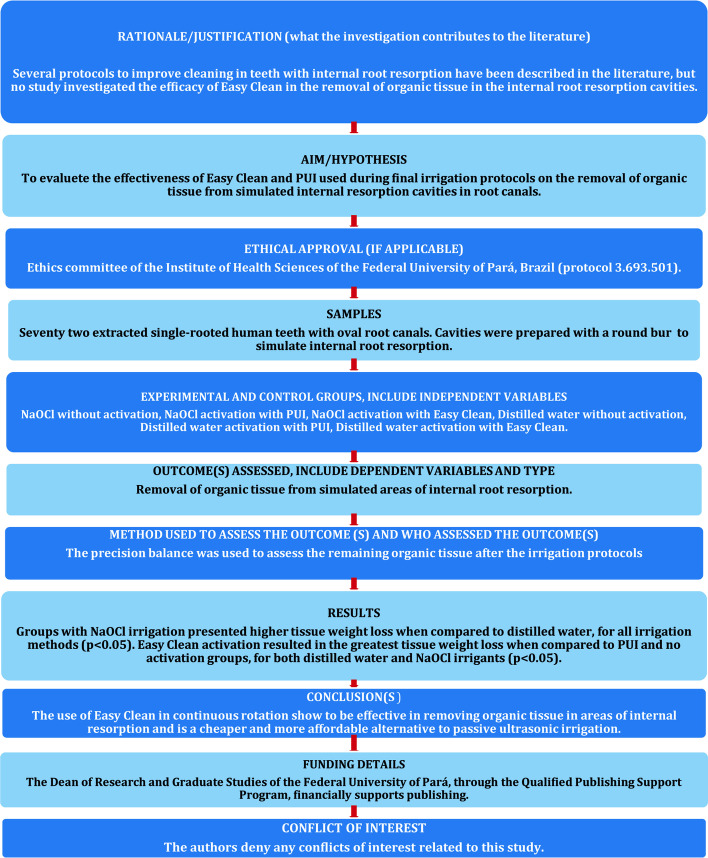
Preferred Reporting Items for Laboratory studies in Endodontology (PRILE 2021) flowchart

Finally, the canals were flushed with 2 mL of distilled water to prevent the irrigants from having prolonged effects. Paper points were used for drying the root canals. The roots were disassembled, and the remaining bovine tissue inside the resorption cavities was gently blotted dry using absorbent paper (Fig. 1f). The specimens were weighed three times, and the arithmetic mean was calculated and recorded. The major steps involved in this methodology are summarized in the PRILE 2021 flowchart (Fig. 2).

### Statistical analysis

The statistical analysis was performed using the mean difference between pre and post-irrigation weights. Data were analyzed for normal distribution using the Shapiro Wilk test and homogeneity of variance with Levene’s test. Two-way ANOVA was performed to analyze the influence of the two factors (irrigant and activation method) and their interactions on organic tissue removal. Post hoc multiple comparisons were performed by using the Tukey test (*p* < 0.05) (SPSS 15.0 statistical package; SPSS Inc., Chicago, IL, USA).

## Results

There was no significant difference in the initial standard weight – baseline conditions – of the different tested groups (*p* > 0.05). None of the experimental protocols totally removed the bovine tissue. Groups with NaOCl irrigation presented higher tissue weight loss when compared to distilled water, for all irrigation methods (*p* < 0.05). Easy Clean activation resulted in the greatest tissue weight loss when compared to PUI and no activation groups, for both distilled water and NaOCl irrigants (*p* < 0.05). However, no differences were observed between PUI and no activation groups, in neither of the tested solutions (*p* > 0.05). These results can be observed in Table 1. Means tissue weight values pre and post-irrigation can be observed in Table 2.

**Table 1:** Means and standard deviations of % reduction in tissue weight values

Irrigating solution	No activation	Easy Clean	PUI
Distilled water	33.4 ± 7.3^Ab^	42.0 ± 9.0^Bb^	33.3 ± 10.3^Ab^
NaOCl 2.5%	38.8 ± 9.8^Aa^	45.5 ± 9.4^Ba^	37.7 ± 6.8^Aa^

Different letters indicate statistically significant differences (*p* < 0.05). Capital letters were used to compare groups in rows (irrigation methods), and lowercase letters were used to compare groups in columns (irrigating solutions) separately

**Table 2:** Means tissue weight values pre and post-irrigation (g)

Irrigating solution	No activation	Easy Clean	PUI
	Pre/post-irrigation		
Distilled water	0,0035/ 0,0023	0,0035/ 0,0020	0,0035/ 0,0023
NaOCl 2.5%	0,0033/ 0,0023	0,0034/ 0,0018	0,0035/ 0,0021

## Discussion

Eliminating granulation tissue in teeth with internal root resorption is fundamental, as the tissues can provide substrates for microorganisms and lead to endodontic treatment failure [1]. The results of the present study revealed that the use of Easy Clean mechanical activation device in continuous rotation was more effective than the use of PUI in eliminating tissue samples from simulated internal resorption cavities. Therefore, the null hypothesis tested were rejected. In fact, Easy Clean showed better results than those of PUI and conventional irrigation even when the irrigant was distilled water.

The better results obtained by Easy Clean might be explained by the device's action mechanism that results in the agitation of the irrigating solution and the direct action of its blades on the root canal walls. In the present study Easy Clean was used in continuous rotation, as a previous study showed better cleaning results than using it in a reciprocating motion [9]. Although no studies are analyzing the efficacy of Easy Clean in the removal of organic tissue in simulated internal resorption defects, the use of XP-endo Finisher resulted in the greatest tissue weight loss compared to the PUI from artificial internal root resorption cavities [21]. Kato et al. [12] also showed superior effectiveness in removing debris from the apical third after using Easy Clean in comparison with PUI. Nevertheless, other studies showed similar efficacy of both methods in the removal of calcium hydroxide in oval root canals [11], removal of remnants of filling material [17], reduction in bacterial load [18, 19], and penetration of the irrigating liquid into simulated lateral canals [16].

Despite the lower elimination of tissue samples for PUI in the present study, a recent systematic review showed better results in cleaning the root canal and isthmus during endodontic therapy when using PUI compared to that conventional irrigation [25]. In the present study, agitation was performed with instruments located 2 mm from the apex, and the results could be different if the ultrasound tip was close to the internal resorption, as the shear stress is higher around the tip [26]. This points to a limitation of the study, although there was a need to standardize the use of both instruments at the same working length. Furthermore, although there are no studies evaluating the influence of irrigation volume compared to the use of PUI in the removal of organic tissue, Zorzin et al*.* [27] verified that irrigation volume was more effective than activation for the removal of calcium hydroxide. In fact, the volume of irrigant solution increases the tissue dissolution capacity of NaOCl [28] and further studies should evaluate this effect in internal resorption defects.

There was a significant difference between the use of distilled water and NaOCl, in line with previous studies that demonstrated the tissue dissolution capacity of NaOCl [3, 29–31]. It is essential to mention that the difference between the irrigation solutions tested could be higher if the exposure time to NaOCl were longer, as previous studies demonstrate a time-dependent effect on organic tissue dissolution by NaOCl [32, 33]. Furthermore, Stojicic et al. [32] found that increasing solution concentration, temperature, and the addition of surfactants improved the tissue-dissolving effectiveness of NaOCl even 50-fold. It has been suggested that the presence of dentine debris favors rapid consumption of the free chlorine of NaOCl, thus affecting its tissue dissolution [34]. In the present study, the activation of the solution was performed only during the final irrigation, which may have removed less dentine debris. Future studies should assess the need for substance activation during instrumentation. Additionally, further studies should investigate the influence of instrument kinematics during chemical–mechanical preparation of the root canals system, since there are currently no data comparing the different kinematics on the organic tissue removal from internal resorption defects.

It is important to highlight that the present study was performed under laboratory conditions, and in clinical situations, the results could be different. Bovine meat was used because of its large availability, uniform composition that resembles pulp tissue and ability to be cut to a similar size and weight, which allows randomization [34]. Simulating pulp tissue is a widely used method in Endodontic research since the human pulp is difficult to source and standardize [21, 30, 35]. However, in clinical practice the pulp tissue may encounter more resistance to be removed from the root canal walls, which can be considered a limitation of the present research. It is also important to emphasize that in a clinical setting, dental pulp tissue would be partially dissolved during cleaning and shaping procedures performed before the final irrigation protocol. However, in this study, organic tissues in simulated cavities were directly treated with irrigation protocols for standardization purposes.

## Conclusions

The results demonstrated the importance of the agitation process during root canal irrigation. The use of Easy Clean in continuous rotation show to be effective in removing organic tissue in areas of internal resorption and is a cheaper and more affordable alternative to PUI.

## Data Availability

The datasets used and analysed during the current study are available from the corresponding author on reasonable request.

## Abbreviations

NaOCl: Sodium hypochlorite
PUI: Passive ultrasonic irrigation
WL: Working length
